# Attitudes of Asian and Polish Adolescents towards the Use of Ecological Innovations in CPR Training

**DOI:** 10.3390/jcm12216939

**Published:** 2023-11-05

**Authors:** Filip Jaskiewicz, Dariusz Timler

**Affiliations:** Emergency Medicine and Disaster Medicine Department, Medical University of Lodz, 90-419 Łódź, Poland; dariusz.timler@umed.lodz.pl

**Keywords:** medical education, cardiopulmonary resuscitation, ecology

## Abstract

Background: The potential use of manikins made of environmentally friendly materials (biodegradable or easily recycled) could be a milestone in promoting cardiac arrest awareness and mass resuscitation training without the threat of generating large amounts of unprocessable waste. The main aim of the study was to compare the attitude of young adults from Asia and Poland towards cardiopulmonary resuscitation training forms and to evaluate the innovative concept of an ecological resuscitation manikin; Methods: This was a survey-based study conducted during two events in Thailand and Poland in 2023; Results: A total of 226 questionnaires were included in the final analysis. Asian respondents were significantly more likely to choose traditional training than Polish participants (78% vs. 58%, respectively). A manikin that is mainly biodegradable was the most common choice across the entire study group. Young Asians were significantly more likely to choose a traditional stationary course, while Polish respondents were highly significantly more likely to opt for hybrid training (online with practical training provided at the student’s home). Conclusions: In the total study group, young people from Poland and parts of Asia are most likely to participate in traditional on-site instructor-led training, but a comparison across groups showed a significant tendency for young Poles to choose a hybrid training option, i.e., a combination of online and hands-on training. Despite some differences, both study groups showed a strong interest in pro-environmental behavior and the use of more ecofriendly solutions than previously used in resuscitation training.

## 1. Introduction

Despite the progress made, mortality in out-of-hospital cardiac arrest (OHCA) remains very high [1,2]. While earlier studies mainly focused on the clinical aspect of sudden cardiac arrest (SCA) and cardiopulmonary resuscitation (CPR), growing research has focused in recent years on the implementation of guidelines and effective CPR training [3,4,5,6,7,8,9]. Recent guidelines from the European Resuscitation Council (ERC) and the American Heart Association (AHA) have also devoted considerable attention to the issue of human factors in SCA rescue training [9,10]. 

With regard to survival, the undertaking of CPR by an SCA bystander plays a crucial and undeniable role in increasing the chances of survival. Moreover, it is one of the few factors that also contribute to a good neurological outcome [9,10,11,12,13,14]. Therefore, some of the ERC guidelines that focus on education emphasize not only the role of effective, high-quality CPR training, but also measures to improve CPR readiness and confidence in SCA bystanders [13,14]. Numerous studies indicate that these elements are positively influenced by hands-on training [3,4,5,6,7,8,15]. From this point of view, all efforts to increase the availability of practical CPR training are extremely important [16]. This includes both traditional courses and short mass training programs to promote first aid and CPR [17,18]. Considering the above issues, it seems important to seek technical solutions to increase the availability of reproducible CPR training for all populations [9,10,11,12,18,19,20]. To this end, it is necessary to reduce costs while maintaining the effectiveness of practical training. Commercial low-cost CPR manikins are well-known. However, researchers and developers continue their search for other solutions that may be better, cheaper or simply alternative [21,22]. Unfortunately, the low price of a product is also associated with its shorter life cycle. Moreover, the materials from which mannequins are made are not always environmentally friendly and generate waste. The potential use of manikins made of environmentally friendly materials, such as cardboard or coconut fiber, which are biodegradable or easily recycled, could be a milestone in promoting SCA awareness and mass CPR training without the threat of generating large amounts of unprocessable waste. 

The subject of the presented study is novel since it connects issues highly important from the perspective of human life and public health with a broader perspective related to ecology and the environmental safety of the planet. Research is increasingly involved in assessing the attitudes of different social groups towards the daily use of organic products [23,24], including those potentially used for medical purposes [25,26]. However, studies on environmental issues related to emergency medicine and medical education are very scarce. This prompted us to assess the opinions of first-time users, who were presented with an innovative environmentally friendly resuscitation training manikin.

## 2. Aims

The main objective of the study was to compare the attitudes of young adults from different parts of Asia (AS group) and Poland (PL group) towards CPR training and to evaluate a novel concept for a CPR training manikin made of environmentally friendly materials. 

The specific objectives were to assess and compare the following variables across study groups:

- preferred form of CPR training;
- preferred manikin in terms of materials used for its construction;
- respondents’ attitudes to a manikin made of ecological materials and its usefulness in mass CPR training.

## 3. Materials and Methods

### 3.1. Study Setting

**Figure 1:**
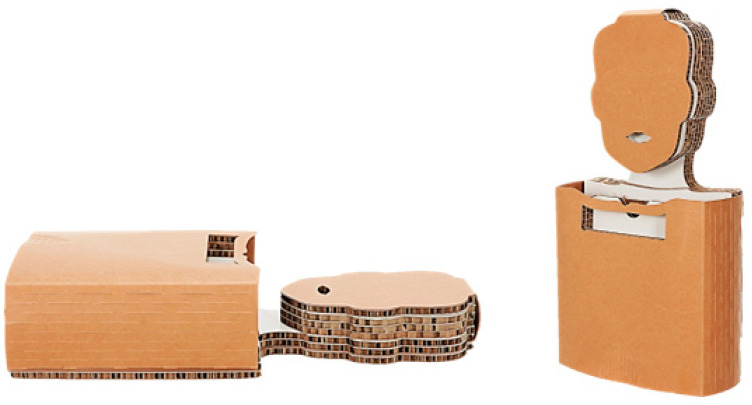
ECO CPR—Ecological manikin for CPR training made of cardboard, coconut fiber and steel springs, used in the study.

**Figure 2:**
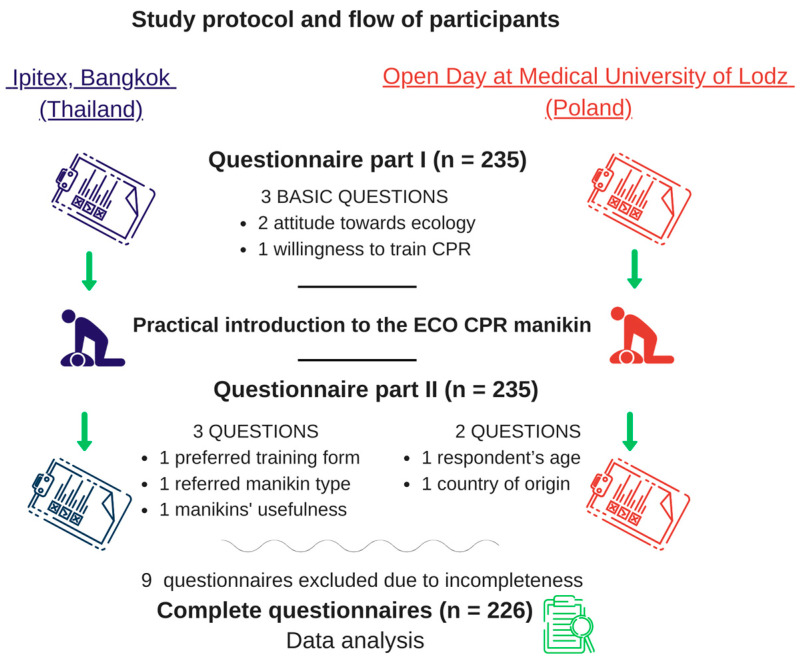
The study protocol and participant flow. Ipitex—International Intellectual Property, Invention, Innovation and Technology Exposition.

This survey-based study was conducted during the first official presentation of the ecological CPR training manikin (ECO CPR, Medical University of Lodz, Poland, Figure 1), which was held at the International Intellectual Property, Invention, Innovation and Technology Exposition (Ipitex) in Bangkok (Thailand) between 2 February 2023 and 6 February 2023 and during the Open Days of the Medical University of Lodz on 15 March 2023 (Poland). Each participant had the opportunity to familiarize themselves with the manikin and its technical capabilities, and to assess its functionality in practice. The manikin familiarization procedure presents the possibilities of chest compressions, opening the airway by lifting the chin and giving rescue breaths. The study protocol and participant flow are presented in Figure 2. The study was approved by the Bioethics Committee at the Medical University of Lodz (number RNN/54/23/KE).

### 3.2. Participants

All participants of the above-mentioned events were eligible to take part in the study. Since the study aimed to assess first impressions of the manikin in the young adult population, ages < 15 years and >30 years were the exclusion criterion in subsequent data selection. 

### 3.3. Data Sources/Measurement

The survey used an online questionnaire (File S1). Microsoft Forms software (ver. 18.2306.1061.0, Microsoft Corporation, Redmond, Washington, USA) made available via a QR code was used. Participation in the survey was voluntary. Questions regarding attitudes towards the environmental safety of the planet, the use of eco-friendly products in daily life, a willingness to receive CPR training and its form were asked prior to the presentation of the environmentally friendly CPR training manikin. Questions on the choice of manikin on which respondents would like to train and an overall assessment of attitudes towards the low-cost, cardboard manikin were answered by the study participants after familiarizing with the manikin’s characteristics and capabilities.

The process of creating and evaluating the questionnaire was as follows: 

Stage 1.—Literature review to identify questions used in research on similar topics, including literature reviews, original articles, guidelines and international recommendations.

Stage 2.—Analysis of questions used in other researchers’ questionnaires and their critical verification in relation to consistency with the aims of the presented study.

Stage 3.—Preparation of a list of questions appropriate to verify the chosen aims of the study. 

Stage 4.—Evaluation of the content by subject matter experts (*n* = 3). 

Stage 5.—Presentation of the questionnaire in a pilot form to 21 respondents to assess whether:

- It is comprehensible for respondents;
- It is interpretated in the same way by all the respondents;
- Any aspect of the questionnaire suggests researcher’s bias.

Stage 6.—Completing the evaluation of the questionnaire.

### 3.4. Statistical Analysis

Due to the nature of the study and the size of the potential general population of young people in Poland and Asia, no analysis of the minimum sample size was performed with reference to the entire population, as the values resulting from this calculation would have been unattainable. The sample size was therefore calculated with reference to the population of young people who were thoroughly acquainted with the ecological manikin prototype at both events. Using G* power software 3.1.9.2, at least 89 participants from the AS group and 131 from the PL group were needed to achieve power = 0.95 and error = 0.05. To provide a safety margin in case of missing data or non-participation, the minimum size of the study group was increased to 95 and 140 participants, respectively. Statistical analyses were conducted using the statistical package PQStat version 1.8.4.152. Elements of descriptive statistics were used to determine mean (x), minimum (Min), maximum (Max), percentage (%) and standard deviation (SD). The results for responses to the questions “In scale 1 to 5 how the ecological safety of our planet is important for you?” and “How do you rate the manikin in terms of usefulness in CPR training and mass CPR training?” were compared using the Mann–Whitney U test, depending on the group. The results of the answers to the questions: “Do you think it is important to use ecological products in everyday life?”, “What type of CPR course would you choose if you had a choice?” and “What type of manikin for CPR training would you choose if the cost of training and the functionality of the manikin were similar?” were compared using the chi2 correlation test and the Fisher’s exact test, depending on the group.

A *p* value < 0.05 was considered significant and a *p* < 0.01 was considered highly significant.

## 4. Results

**Figure 3:**
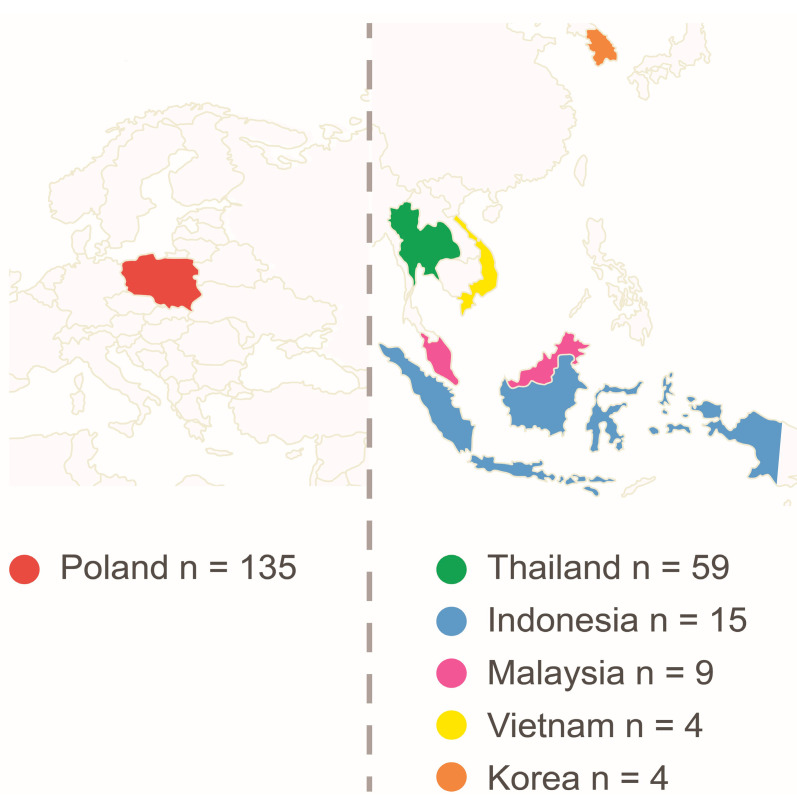
Country of origin and the number of participants in each group of respondents.

A total of 235 participants took part in the study. After rejecting nine questionnaires due to incomplete data, 226 questionnaires were included in the final analysis. There were 91 respondents (AS group) from Asia and 135 from Poland (PL group). The mean age of the respondents was 18.9 ± 3.4 years and did not differ significantly between the groups (AS group—x = 18.6 ± 3.4 vs. PL group—x = 19.5 ± 3.5, respectively). Respondents in the AS group came from different countries (Figure 3).

The scores on the five-point scale in response to the question on “How the ecological safety of our planet is important for you?” differed highly significantly between the groups and were higher in the AS group (x = 4.8 ± 0.4 vs. 4.4 ± 0.8, respectively, *p* < 0.01). Analysis of the respondents’ answers to the question “Do you think it is important to use ecological products in everyday life?” also showed a statistically significantly higher frequency of an affirmative answer among the Asian respondents (AS group 100% vs. PL group 91%; *p* = 0.003). The vast majority of respondents would like to take part in CPR training in the future. In this case, there were no significant differences between the study groups (Table 1).

**Figure 4:**
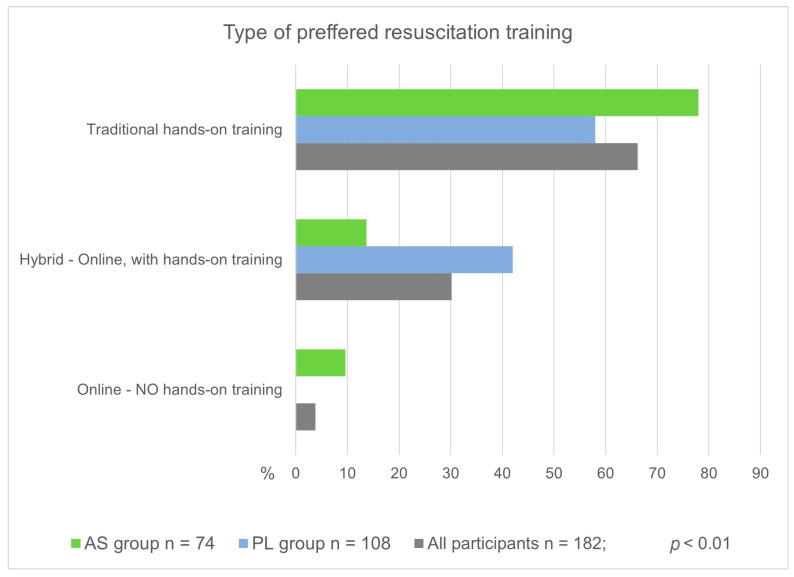
Frequency of responses to the question regarding the form of CPR training preferred by participants.

With regard to the form of CPR training among respondents who expressed their willingness to participate in such course in the future, the choice of traditional, stationary training with exercises (hands-on training) clearly dominated across the entire study group and the individual AS and PL groups (Figure 4). However, further analysis revealed highly statistically significant differences in the choice of the form of training between the AS and PL groups (*p* < 0.01). AS respondents were significantly more likely to choose traditional training than PL participants (78% vs. 58%, respectively). None of the PL respondents expressed a preference for exclusively online training, which was chosen by 9.6% of AS respondents (Figure 4).

**Figure 5:**
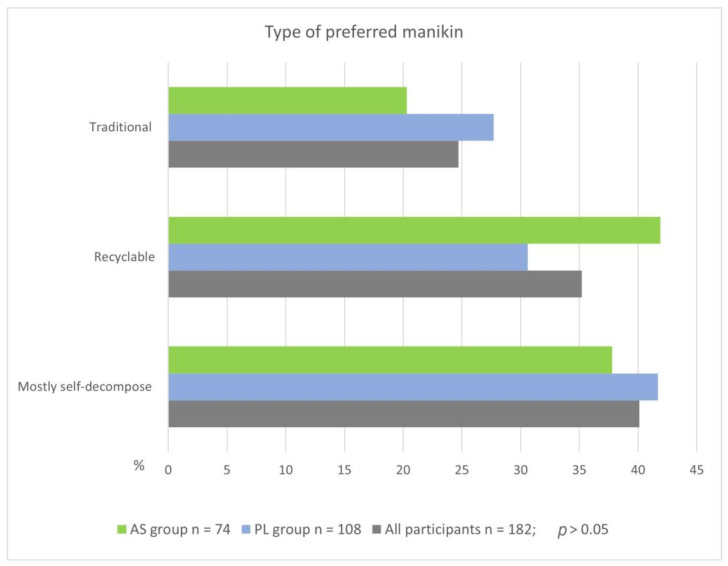
Frequency of responses to the question on the choice of type of manikin for CPR training.

In contrast to the preferred form of training, the distribution of responses to the question “What type of manikin for CPR training would you choose if the cost of training and the functionality of the manikin were similar?” did not differ significantly between Asian and Polish respondents (*p* = 0.250). A manikin, which is mainly biodegradable, was the most common choice across the entire study group, whereas a traditional manikin made of mostly non-recyclable plastics was the least frequent choice (Figure 5).

**Figure 6:**
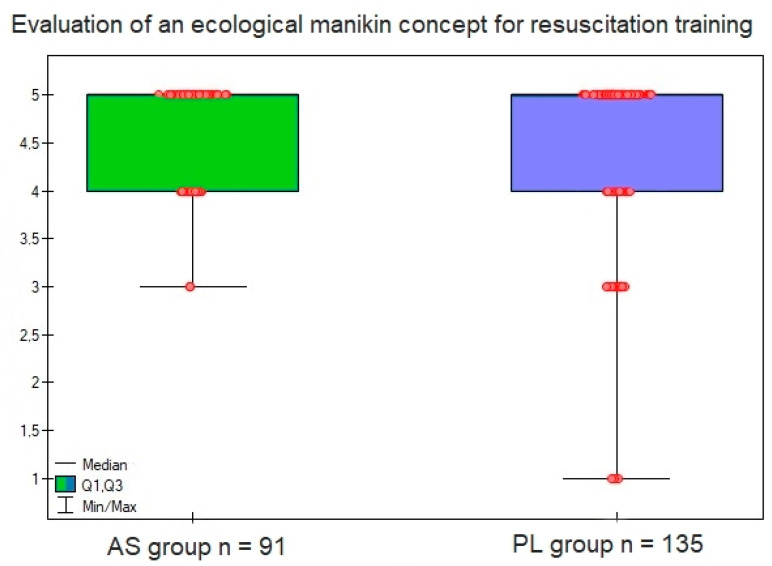
Evaluation of the concept of an ecological CPR training manikin using the example of the prototype presented to the study participants.

In the last question, the respondents rated on a five-point scale to what extent they liked the concept of using an ecological manikin for CPR training and mass CPR training based on the presented prototype made of cardboard, coconut fiber and steel springs. Although the mean score was higher for Asian respondents, data analysis showed no significant differences—AS group x = 4.6 ± 0.5 vs. PL group 4.5 ± 0.8, *p* = 0.713, respectively (Figure 6).

## 5. Discussion

In view of the ever-low survival rate of out-of-hospital cardiac arrest patients, it is highly important to look for factors and implement solutions that can increase the chances of survival [1,2,12]. The most recent ERC and AHA guidelines and numerous scientific papers point to the importance of CPR training and coaching in the general population [9,10,11,12]. Undertaking CPR by SCA bystanders is undeniably associated with increased chances of survival and better neurological outcomes [12,13,14]. The main challenges to be faced in this context are not only to find and apply a method of effective CPR training, but also to increase the willingness and readiness of witnesses to undertake CPR. The latest ERC and AHA guidelines [1,2,9,10,11,12] pay particular attention to the human factor as necessary to initiate the chain of survival [27]. For the first time the recommendations focus attention on identifying the needs of trainees, selecting training programs to match the characteristics of trainees, and including a conversation about shaping the willingness of potential witnesses to undertake life support measures in the CPR course topics to such an extent. The ERC education guidelines emphasize that the teaching of practical skills alone is not necessarily related to the willingness to undertake CPR in a real-life situation [11,12,13]. Indeed, many factors influence this final outcome. This is described in some detail in the paradigm by Panchal et al. [28]. He points out that a number of modifiable factors influence the bystander’s decision to initiate CPR. In this context, it seems important to assess needs and to actively shape the way in which CPR courses are delivered. This applies both to the way the course is organized and to the training and teaching equipment used, from the modern form of remote communication to training manikins for the practice of chest compressions and rescue breaths. 

The present study therefore undertook a comparative analysis of the preferred forms of CPR training among young people from Asia and Poland. Respondents’ opinions on the use of a novel CPR training manikin constructed from eco-friendly materials, i.e., cardboard, coconut fiber and steel springs, in terms of usefulness in CPR training and mass CPR training were also assessed. In order to attain a better sense of the benchmarks and to obtain more reliable answers to this question, the participants were also asked two other questions about their attitudes towards and opinions on environmental issues. Of course, obtaining answers to these questions was not intended to thoroughly compare the respondents’ approaches to ecology, but only to highlight their general characteristics on the topic. 

The results of the survey showed that young people from both Poland and Asia declare concern for environmental welfare. These results are interesting in that the two groups of respondents come from very different cultural, economic and social backgrounds. Since these respondents were young adults, who were just entering their productive years and would themselves be responsible for shaping the attitudes of future generations, the results obtained may seem all the more optimistic from a future perspective [24,25,26]. Statistical analysis of the data showed, however, that it is the Asian respondents who, on the five-point scale, highly significantly rated their concern for the ecological safety of the Earth higher, with a mean x of 4.8 ± 0.4 vs. 4.4 ± 0.8, respectively (*p* < 0.01). The Asian respondents were also highly statistically significantly more likely to indicate that they believe it is important to use organic products in their daily lives. All of the young Asians surveyed agreed that this was important for them. Although concern for this issue and product choices among the Polish respondents were also high, this aspect was not important for almost one in ten respondents. 

The evaluation of the concept and the ecological manikin in terms of usefulness in CPR training and mass CPR training showed a higher score on a five-point scale for respondents from the Asian group (x = 4.6 ± 0.5 vs. the PL group 4.5 ± 0.8, *p* = 0.713), which also seems to be consistent with these declarations. In this case, however, the difference was not statistically significant. Statistically significant differences were also not found between the Polish and Asian groups in terms of their responses to the question about which manikin, from the perspective of the materials used in its construction, they would like to train on. Among all participants in the study, the most commonly selected type of manikin was (in order): mainly biodegradable, recyclable, traditional—which, according to the available specifications of some manufacturers, is commonly made of materials such as: polyvinyl chloride (PVC), low density polyethylene, rubber, silicone, etc., [29,30]. This choice is consistent with the declarations of concern for the ecological safety of the Earth and the use of green products in daily life from the previous questions. Asian respondents were the most likely to choose a manikin whose components can be recycled after being placed in appropriate containers, while respondents from Poland preferred a manikin that is mainly biodegradable and only part of it is recyclable. 

Obviously, this question was only answered by respondents who expressed their willingness to participate in CPR training in an earlier survey question. Although, with such a small size of the study group in relation to the entire population of young people in Asia and Poland, the answers obtained do not give a full picture of the willingness to participate in CPR training. Just as is the case with the first two questions from the questionnaire, they helped to bring us closer to understanding the respondents’ attitude to CPR education. The rate was 81.3% in the AS group vs. 80% in the PL group, respectively, and the difference was not statistically significant. When compared to the percentage of people who have ever attended a CPR training course, based on reports from other researchers, which vary significantly from country to country: 82.9% in Korea [3], 34.8–37.6% in China [31,32], 45.7% in Taiwan [33], 53% in Crimea [34], 76% in Sweden [35], 89% in Germany [36], and 90% in Norway [37], such a high rate of willingness to attend CPR training may bode well for the future. Especially since the present study was conducted after the SARS-CoV-2 pandemic, which, as reported by many researchers, may influence a change in attitudes and behavior toward bystander cardiopulmonary resuscitation in OHCA [38,39,40,41,42,43]. 

Despite the relatively small size of the study group in presented study, this can lead us to believe that populations of young people, regardless of their country or region of origin, will be more willing to be trained in CPR and thus will contribute to an increasing the rate of resuscitation attempts in OHCA, thereby increasing survival with good neurological outcomes [2,9,10,11,12]. The latest systematic review by Daud et al. concluded that there are six factors influencing community willingness and training is one of them. Also, the study reveals also that the urgent measures that need to be taken are ongoing and frequent CPR and AED intervention education programs, as well as the formulation of policies and laws to require the community’s implementation of CPR and AEDs [44].

In the present study, interesting results were obtained regarding the mode of training the study participants would like to receive. A residential course with hands-on training was the most common option across the entire study group. A comparative analysis of the Asian and Polish groups showed a highly statistically significant difference. Young Asians were significantly more likely to choose a traditional stationary course, while Polish respondents were highly significantly more likely to opt for hybrid training, in which classes are held remotely (online), but practical training is provided at the student’s home. Due to the need to keep the questionnaire as short as possible and tailored to the type of events during which it was conducted, the reasons for such choices were not analyzed. 

At the same time, regardless of the form chosen by the participants, it should be borne in mind that any of them may be beneficial even if they are dependent on the target group and technical, organizational, and financial capabilities [8,18,45]. A systematic review by Allan et al. observed that innovative approaches, such as peer and self-based CPR training are equally effective as instructor-led methods when teaching schoolchildren [46]. González-Salvado et al., who performed a systematic review of the literature on training adult laypeople in CPR, emphasized that instructor-led hands-on training supported by feedback devices is superior to other methods but still the fact is that any training already contributes to self-evaluation or the willingness to use skills, with overall positive results regardless of the teaching method used [8]. At the same time, we should remember the role of the human factor in the teaching implementation process, emphasized by the ERC guidelines. So that the training process and the form of its organization are adapted to the conditions and the participants themselves [11,12]. 

Therefore, in the current study, we wanted to find out what the attitude of young people is towards the preferred form of training, the type of manikin, and to assess the possibility of using the presented prototype for CPR training and mass CPR training. Especially since many concepts are currently being developed. Some of these strive for extremely high training credibility, which may involve the use of non-ecological materials. Others transfer participants, using electronic systems and computers, into virtual reality or augmented reality. Our study aimed to check whether, in addition to such sophisticated tools, simpler but fully ecological ones could potentially gain recognition among young people. Especially since the potential use of ecological and low-cost solutions in resuscitation education can potentially be applied in many ways. In addition to standard training in small groups, this could create new opportunities for mass CPR training or hybrid methods (online with hands-on training). This last example may be particularly applicable in exceptional situations such as a lockdown during a pandemic [38,39,40,41,42,43]. It potentially enables online education conducted by an instructor combined with low-cost hands-on training. The ecological mannikin itself, after being used by household members, would not constitute non-recyclable waste.

It should also be remembered that, in relation to improving the quality of care in SCA and the implementation of CPR skills, measures and solutions known and obvious in one region may be unavailable in another region of the world. An International Liaison Committee on Resuscitation statement on cardiopulmonary resuscitation in low-resource settings reveals primary action points on that matter. In the education category, the statement suggests considering alternative teaching approaches such as distance, virtual, hybrid, and blended-learning methods, and low-cost mannequins [47]. Most importantly, attempts to combine the implementation of resuscitation guidelines in the form of mass training to reach entire communities with the simultaneous promotion of environmentally friendly behavior and inexpensive solutions are increasingly being put into practice but remain rather rare. CPR manikins made of PET bottles or the cardboard eco-friendly manikin “CANNE—Self-directed CPR learning” developed at Umeå University, different from the one presented in this study, are examples of such educationally successful measures [22,48,49,50].

Future research should focus on the difficult task of finding the right recipe to meet diverse needs. Including, among other things: effective training methods and techniques and equipment enabling their implementation, and methods of organizing courses/mass training sessions so that they reach entire societies regardless of age group and economic balance. The present work also draws attention to additional aspects of that challenge, such as environmental sustainability and the human factor in shaping the method of training and the equipment used. Educators, designers, producers as well as researchers should also take into account whether a given training method will be well received by the target participants themselves. The attitude and general emotions experienced during the process of acquiring knowledge are important factors in remembering what we learn. 

Despite some differences, even those statistically significant, the results of the survey among young people from Asia and Poland can be considered to be very optimistic. Their opinions may differ due to cultural, economic, religious, social or political factors, but in these most important respects, they reinforce a belief in progress in terms of awareness in two important dimensions: saving lives and striving for practical application of eco-friendly solutions, even in unusual applications.

## 6. Limitations

The relatively small number of respondents representing the comparison groups in the analyses was the main limitation of the study. The nature and aim of the study, as well as the unusual issue addressed meant that the analysis of the minimum size of the respondent groups had to be limited to participants of the two events who had the opportunity to closely familiarize with the ecological manikin. Additionally, the respondents may not be fully representative of the age group since their participation in the events at which the survey was conducted may have influenced their general preferences, opinions or attitudes towards the issues raised in the questionnaire. The questionnaire itself, being short and simple, was therefore not fully evaluable, which may have limited its power. In the case of the AS group, the diverse backgrounds of the respondents in terms of their country of origin may have been an additional limitation affecting the heterogeneity of the group.

## 7. Conclusions

In the total study group, young people from Poland and parts of Asia are most likely to participate in traditional on-site instructor-led training, but a comparison across groups showed a significant tendency for young Poles to choose a hybrid training option, i.e., a combination of online and hands-on training. Despite some differences, young people from Poland and Asia showed a strong interest in pro-environmental behavior and the use of more eco-friendly solutions than previously used in CPR training.

## Figures and Tables

**Table 1:** Frequencies of reporting a willingness to take part in CPR training in the future among study participants.

	AS Group N = 91 (%)		EN Group N = 135 (%)		All Participants N = 226 (%)		*p*-Value
Would you like to take part in a CPR training in the future?	YES 74 (81.3)	NO 18 (19.7)	YES 108 (80.0)	NO 28 (20.0)	YES 182 (80.5)	NO 46 (19.5)	>0.05

## Data Availability

Not available.

## Supplementary Materials

The following supporting information can be downloaded at: https://www.mdpi.com/article/10.3390/jcm12216939/s1, File S1: Survey Questionnaire Form.
