# Supplementary material for: Attitudes of Asian and Polish Adolescents towards the Use of Ecological Innovations in CPR Training
Source: J Clin Med. 2023 Nov 5;12(21):6939. doi: 10.3390/jcm12216939 (PMC10648462; doi:10.3390/jcm12216939)
Supplement: Supplementary file 1 [file jcm-12-06939-s001.zip › jcm-2645805-supplementary.pdf]

File S1: Survey Questionnaire Form

PART I

Please take part in the anonymous, voluntary survey and help us develop knowledge about resuscitation training and its new forms! It will only take a few minutes.

1. In scale 1 to 5 how the ecological safety of our planet is important for you?

   (1—Not important at all; 5—Very important)

   □ 1
   □ 2
   □ 3
   □ 4
   □ 5
2. Do you think it is important to use ecological products in everyday life?

   □ Yes
   □ No
3. Would you like to take part in a practical training in cardiopulmonary resuscitation (CPR)?

   □ Yes
   □ No

PART II

1. What type of CPR course would you choose if you had a choice?

   □ Online—NO hands-on training
   □ Traditional hands-on training
   □ Hybrid—Online with hands-on training
2. What type of manikin for CPR training would you choose if the cost of training and the functionality of the manikin were similar?

   □ Traditional
   □ Recyclable
   □ Mostly self-decompose
3. How do you rate the manikin in terms of usefulness in CPR training and mass CPR training?

   (1—Very low; 5—very high)?

   □ 1
   □ 2
   □ 3
   □ 4
   □ 5
4. Please enter your age:

   …………….
5. What country are you from?

   …………….
